# Terminase Large Subunit Provides a New Drug Target for Herpesvirus Treatment

**DOI:** 10.3390/v11030219

**Published:** 2019-03-05

**Authors:** Linlin Yang, Qiao Yang, Mingshu Wang, Renyong Jia, Shun Chen, Dekang Zhu, Mafeng Liu, Ying Wu, Xinxin Zhao, Shaqiu Zhang, Yunya Liu, Yanling Yu, Ling Zhang, Xiaoyue Chen, Anchun Cheng

**Affiliations:** 1 Institute of Preventive Veterinary Medicine, Sichuan Agricultural University, Chengdu 611130, Sichuan, China; qzylj_yll@163.com (L.Y.); mshwang@163.com (M.W.); cqrc_jry@163.com (R.J.); shunchen@sicau.edu.cn (S.C.); zdk24@sicau.edu.cn (D.Z.); liumafengra@163.com (M.L.); yingzi_no1@126.com (Y.W.); xxinzhao@sicau.edu.cn (X.Z.); shaqiu86@hotmail.com (S.Z.); yunnyaaliu@163.com (Y.L.); yanling3525@163.com (Y.Y.); zl97451@126.com (L.Z.); chenxy_24@sina.cn (X.C.); 2 Research Center of Avian Diseases, College of Veterinary Medicine, Sichuan Agricultural University, Chengdu 611130, Sichuan, China; 3 Key Laboratory of Animal Disease and Human Health of Sichuan Province, Chengdu 611130, Sichuan, China

**Keywords:** herpesvirus, terminase large subunit, ATPase, nuclease, DNA packaging, antiviral drug

## Abstract

Herpesvirus infection is an orderly, regulated process. Among these viruses, the encapsidation of viral DNA is a noteworthy link; the entire process requires a powered motor that binds to viral DNA and carries it into the preformed capsid. Studies have shown that this power motor is a complex composed of a large subunit, a small subunit, and a third subunit, which are collectively known as terminase. The terminase large subunit is highly conserved in herpesvirus. It mainly includes two domains: the C-terminal nuclease domain, which cuts the viral concatemeric DNA into a monomeric genome, and the N-terminal ATPase domain, which hydrolyzes ATP to provide energy for the genome cutting and transfer activities. Because this process is not present in eukaryotic cells, it provides a reliable theoretical basis for the development of safe and effective anti-herpesvirus drugs. This article reviews the genetic characteristics, protein structure, and function of the herpesvirus terminase large subunit, as well as the antiviral drugs that target the terminase large subunit. We hope to provide a theoretical basis for the prevention and treatment of herpesvirus.

## 1. Introduction

The members of *Herpesviridae* are double-stranded DNA (dsDNA) viruses. According to the International Committee on Taxonomy of Viruses (ICTV), in April 2018 [1], the family *Herpesviridae* was divided into three subfamilies (*Alphaherpesvirinae*, *Betaherpesvirinae*, and *Gammaherpesvirinae*) and an unassigned genus thought to have diverged from a common ancestor 400 million years ago [2,3].

Herpesvirus particles mainly have four components [4,5,6]: the relatively large linear DNA genomes encased within an icosahedral capsid wrapped in a protein layer called the tegument, which is encased in a lipid bilayer membrane called the envelope. In addition, the tegument contains both viral proteins and viral mRNAs. This whole particle is known as a virion, which possess infectivity.

When the herpesvirus is in the process of the lytic replication cycle [7], viral membrane glycoproteins mediate the fusion of the viral envelope with the cell membrane [8,9]. Subsequently, the viral nucleocapsid is released into the cytoplasm, and viral DNA enters the nucleus through nuclear pores [10]. The virus carries out DNA replication, viral gene transcription, capsid assembly, and DNA packaging within the nucleus. Finally, the DNA-containing nucleocapsids pass through the nuclear membrane and the Golgi inclusions in turn to obtain the mature envelope protein and intact outer membrane layer [11]. Mature virus particles are released from the cytoplasm through exocytosis [12].

In HSV-1, the UL15 gene encodes the terminase large subunit, pUL15, which is a nonstructural protein involved in viral DNA cleavage and packaging, along with at least six other proteins (pUL6, pUL17, pUL25, pUL28, pUL32, and pUL33) during virus infection. When any gene is deleted or has a nonfunctional mutation, an accumulation of viral concatemeric DNA and immature capsids will occur within the infected cell nucleus [13,14,15,16,17,18,19]. pUL15, pUL28, and pUL33 are believed to have terminase complex functions that cleave the head-to-tail linked viral concatemeric DNA at a precise location to release unit-length genomes and package the DNA into preformed capsids [20,21,22,23]. This mechanism exists in prokaryotic and eukaryotic dsDNA viruses, such as bacteriophages and herpesviruses [24,25,26,27,28].

## 2. Genome Cleavage and Packaging of the Herpesvirus

In the lytic infection course of herpesvirus, three types of capsid can be separated by sucrose density gradient centrifugation [29,30,31]. A capsids are hollow shells that release the scaffold protein and do not have DNA. These capsids are usually caused by DNA packaging failure. B capsids are angularized capsids that still have the scaffold protein but are not wrapped with DNA and are thought to mature without ever encountering the DNA encapsidation machinery. C capsids are mature capsids that encapsulate the DNA and release the scaffolding. In addition, the procapsid, a precursor form of the A, B, and C capsids, exists in herpesvirus infection. The procapsid is a fragile, largely spherical shell that contains an inner scaffold. The virions first form a procapsid with a scaffold structure in the nucleus. When the DNA is packaged, the scaffold structure gradually disintegrates, and a mature C capsid with a capsule is eventually formed [32,33].

**Figure 1:**
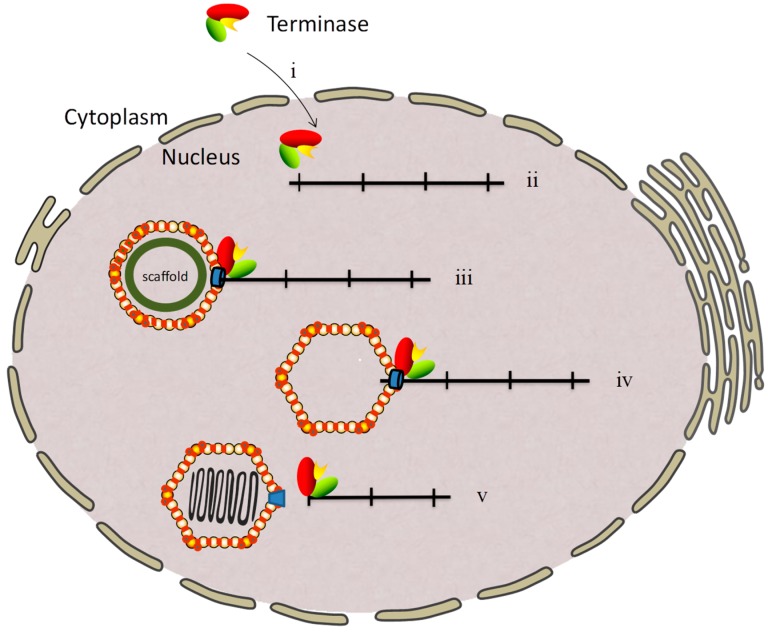
Herpesvirus genome cleavage/packaging. (**i**) Translocation of the terminase complex into the nucleus; (**ii**) viral DNA replication forms head-to-tail linked viral concatemeric DNA; (**iii**) terminase specifically binds the *pac* site, recruits the empty capsid, and cleaves the double-stranded DNA; (**iv**) translocation of a unit-length genome into the capsid; and (**v**) the DNA packaging process is completed by activating the nuclease activity to cut the other end of the individual genome.

During the viral DNA packaging process, the three terminase subunits form a complex in the cytoplasm and then enter the nucleus to perform the functions [34,35]. pUL15 and its homologs are generally considered to be terminase large subunits that function as nuclease to cleave the viral concatemeric DNA [22]. The terminase small subunit, pUL28 and its homologs, plays a role in recognizing and binding to the *pac* elements (*pac*1 and *pac*2) at the end of the free viral genome [36]. The third subunit, pUL33 and its homologs, interacts with the small subunit and enhances the interaction between the large and small subunits. The third subunit is also necessary for releasing individual genomes from viral concatemeric DNA [37]. The predominant model of herpesvirus DNA packaging proposes that the terminase complex binds to the unique portal vertex of procapsids, and the genome is transferred through the portal ring into the capsid in an ATP-dependent manner [13,38,39,40,41]. When a unit-length genome is packaged into the capsid, the nuclease activity of the large subunit is reactivated and cleaves the genome again. The packaging process of the viral genome is thus completed [42,43], and the terminase complex is able to act in additional rounds of cleavage and packaging [41,44,45,46] (Figure 1).

## 3. Characteristics of the Terminase Large Subunit Gene

### 3.1. Terminase Large Subunit Coding by a Splicing Gene

The terminase large subunit of herpesvirus is a highly conserved gene that is referred to by different names in different viruses, such as UL15 in herpes simplex virus 1 (HSV-1), UL89 in human cytomegalovirus (HCMV), and BGRF1/BDRF1 in Epstein–Barr virus (EBV). The terminase large subunit gene is a unique spliced gene in herpesviruses and mainly consists of two exons with a different number of introns. In the α-herpesvirus, the intron generally includes two genes. However, in β- and γ-herpesvirus, the intron generally contains four to five genes (Table 1).

In HSV-1, the terminase large subunit gene usually has two transcripts which are translated into proteins: the full-length protein and the N-terminal truncated protein, known as pUL15 and pUL15.5. Previous studies have shown that the pUL15.5 cannot be detected specifically in any capsid form, whereas the pUL15 is detected predominantly in B capsids and at much lower levels in C capsids and virions [46,47]. In addition, the truncated protein pUL15.5 is not required for viral replication; however, the absence or mutation of full-length UL15 has serious effects on the production of virions [22,35,48]. The Baines lab has shown that there are several truncated forms (80 and 79 kDa) of pUL15 that are found at purified capsids along with the full-length (83 kDa) pUL15. This is important, since they claim that these truncated proteins are the result of proteolytic cleavage which is required for maturation of the viral genome [49]. Therefore, this article focuses on the full-length protein of the terminase large subunit.

### 3.2. Evolutionary Analysis and Sequence Alignment of the Terminase Large Subunit

**Figure 2:**
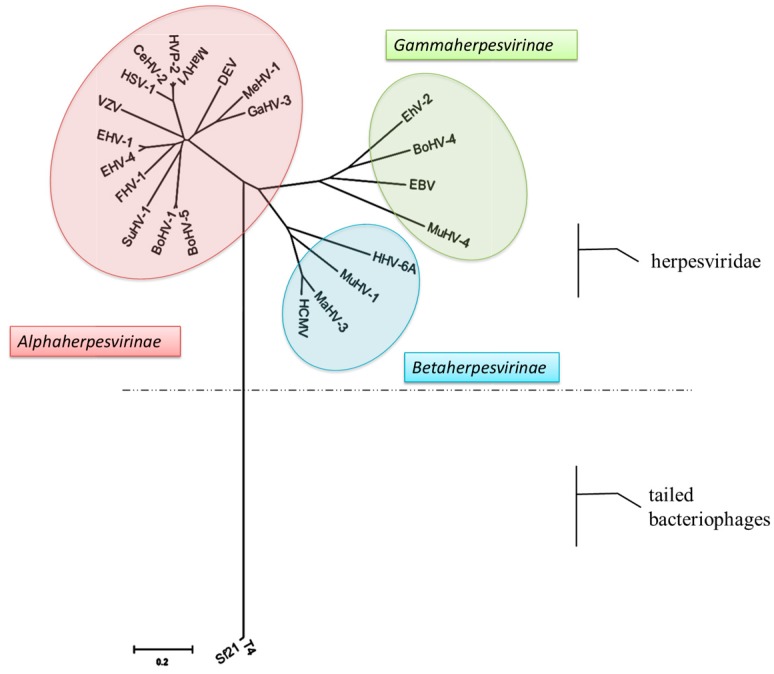
Phylogenetic tree based on the pUL15s sequence of 21 herpesviruses and 2 bacteriophages. The phylogenetic tree was constructed using MAGE7. The pink color represents the α-herpesvirus subfamily, the blue represents the β-herpesvirus subfamily, and the green represents the γ-herpesvirus subfamily. The dotted line above represents the herpesvirus family virus, and below the dotted line represents the phage. The 0.2 below the evolutionary tree represents the evolutionary distance.

**Figure 3:**
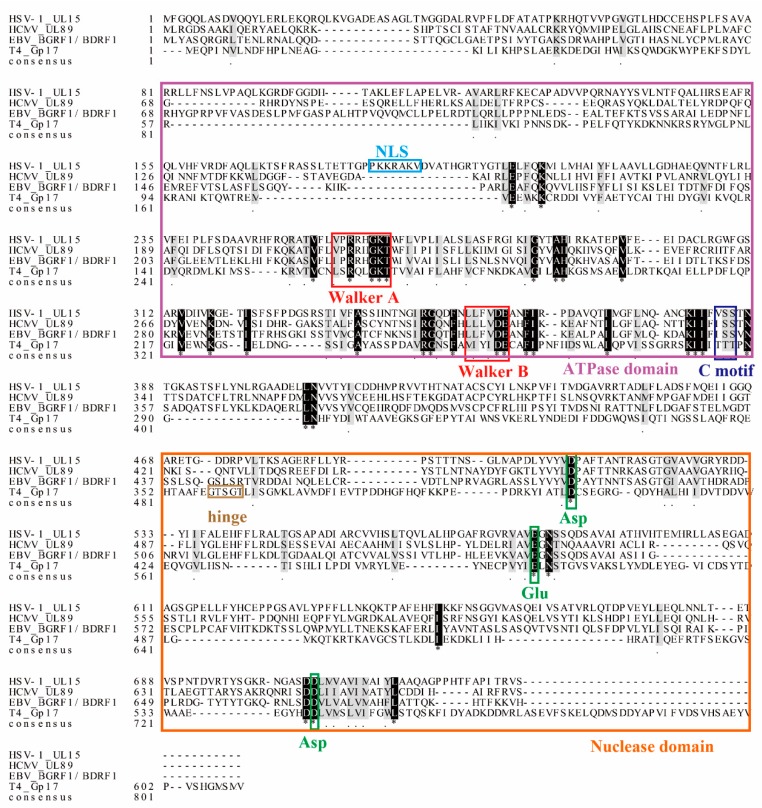
Sequence analysis and functional site comparison of pUL15 and homologs of different subfamilies. The alignment was completed by using MEGA7 and ClustalX2. The purple box represents the amino acid sequence of the N-terminal pUL15 and homologs ATPase activity domain; light blue box represents HSV-1 pUL15 nuclear localization signal (NLS); red box represents the Walker motif; dark blue box represents the C motif; orange box represents pUL15 C-terminal nuclease domain; brown box represents bacteriophage T4 Gp17 Hinge sequence; green box represents the catalytic triplet Asp-Glu-Asp motif. The * means that the amino acids at this site are identical.

To build a phylogenetic tree of the terminase large subunit, we screened 21 pUL15s and their homologs from each herpesvirus subfamily. In addition, two bacteriophage proteins, T4 Gp17 and Sf21 Gp161, were selected as outgroups for the construction of phylogenetic trees, which have been shown to be homologous to herpesvirus pUL15 (Figure 2, Table 1). The phylogenetic tree based on pUL15s was mainly divided into three subfamilies: the α-, β-, and γ-herpesvirus subfamilies, which are consistent with the ICTV-approved virus classification. The results showed that pUL15 may have evolved during the evolution of the species. There is a common evolutionary ancestor of subfamily pUL15s. The study found that pUL15 is a highly conserved protein among the herpesvirus family, which implies the functional significance of this protein. In addition, we could see that the phylogenetic relationship between phage and herpesvirus pUL15 is distant. However, studies have found that they are structurally similar and have the same function [50,51,52]. Therefore, some viruses were chosen from each herpesvirus subfamily and bacteriophage as representatives, and amino acid sequence comparison analysis of pUL15 and its homologs was performed (Figure 3).

As shown in Figure 3, two domains with enzymatic activity, the ATPase and nuclease activity domains, are present in pUL15 and its homologs. Two conserved motifs, Walker A and Walker B, were found in the amino acid sequences of the terminase large subunit proteins in herpesvirus and bacteriophages, and the two conserved motifs are the first to be found in the ATPase domain [25,53,54,55]. Another representative conserved C motif of ATPase has also been found in the amino acid sequences of pUL15 and its homologs. As shown above, pUL15 and its homologs may be involved in ATP hydrolysis. There is also a conserved triplet residue in pUL15s and their homologs, Asp-Glu-Asp, which is similar in amino acid sequence position and protein spatial structure. Previous studies have shown that conserved residues are also present in the nuclease domain of the RNase H structure [56] (Table 2, Figure 3). Mutagenesis studies confirmed that the catalytic motif is essential for enzymatic activity [51,57].

## 4. Structural and Functional Features of the Terminase Large Subunit

### 4.1. Overall Structure of the Terminase Large Subunit

**Figure 4:**
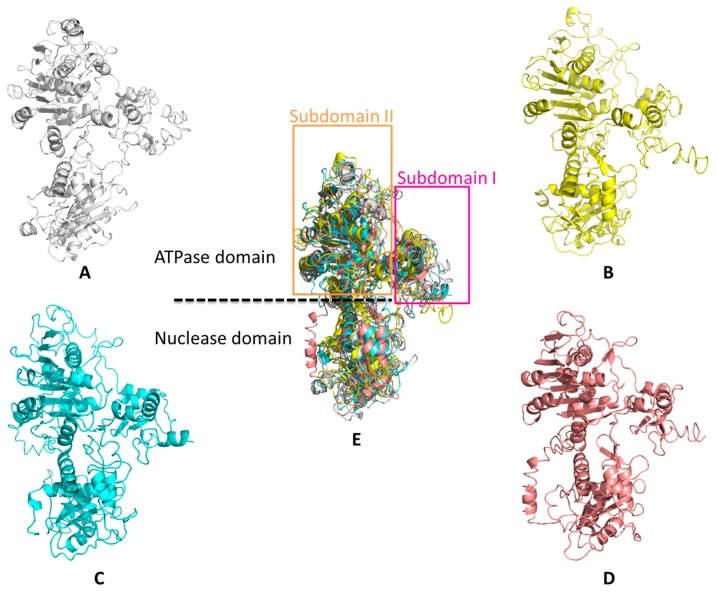
Structural comparison of pUL15 of the four viruses using PyMOL. (**A**–**D**) The pUL15 structures of HSV-1 UL15, HCMV UL89, EBV BGRF1/BDRF1, and T4 Gp17 are represented in gray, yellow, light blue, and pink, respectively. (**E**) Superposition of the HSV-1 UL15C nuclease with large terminase nuclease from HCMV, EBV, and T4. The N-terminal region of the large subunit of the terminase is mainly above the dotted line and mainly contains the ATPase domain. The C-terminal region of the large subunit of the terminase is mainly below the dotted line and mainly contains a nuclease domain.

Previous studies have shown that HSV-1 pUL15 is a catalytic element of the terminase complex and mainly consists of two domains: the N-terminal ATPase domain and the C-terminal nuclease domain [50] (Figure 4A). According to previous reports [58], there is significant flexibility between the ATPase and nuclease domains.

In HSV-1, the folding of the pUL15 C-terminal domain structure (pUL15C) is similar to that of RNaseH, integrases, and topoisomerases, indicating that the catalytic mode of pUL15 is similar to the metal-ion-mediated catalysis of them [50,56]. Each pUL15C molecule contains a seven-stranded β-sheet and six α-helical regions, with parallel and antiparallel strands sandwiched between the helices. The core folds in HSV-1 pUL15C, HCMV pUL89C, RNase H, T4 phage Gp17, and other proteins show a conserved five-stranded mixed sheet arranged as β5-β4-β1-β2-β3, which contains four parallel strands and one antiparallel β2. Negative and positive charges cover the surface of pUL15C [50]. The active site groove has a large negative charge, which is consistent with the recruitment of metal ions for DNA binding and cleavage. In addition, some loops are close to putative nuclease active sites that may interact with DNA substrates, meaning that these loops are flexible and may undergo configurational changes when combined with DNA [50]. A previous study revealed that the C-terminus of the large subunit of the terminase in herpesviruses and phages is mobile and can interact with other components of DNA packaging [50,59,60,61].

The spatial structures of HSV-1 pUL15C and HCMV pUL89C are highly similar, but partial structural elements in pUL15C are different from those in HCMV pUL89 [50,57] (Figure 4). (1) X-ray analysis of the protein crystal structure revealed that the UL15 C-terminus has three protein molecules in the asymmetric unit, but the UL89 C-terminus has four protein molecules. (2) The surface loops L1 and L2 are visible in HSV-1 pUL15C but are invisible in HCMV pUL89C. (3) There is a 31-Å distance between the C-α atom of C-terminal residues of HSV-1 pUL15C and HCMV pUL89C due to the conformational difference between the HSV-1 pUL15C amino acid residues Ala720 to Ile732 and the HCMV pUL89 C-terminal domain. Comparative structural analysis revealed that the two structural elements, β5 and β7, encoded by the two regions Tyr620 to Phe633 and Ile661 to Thr665 in pUL15C exhibited differences in cell RNase H1 and phage homologs. The β5 structure forms an extended surface hairpin structure in pUL15C, but this structure is absent in RNase H1 and SPP1 G2P. Like the β5, the β7 structure is also absent in RNase H1, SPP1 G2P, and P22 gp2 [62,63,64].

Currently, the study of the N-terminal domain of the large subunit of the terminal enzyme mainly concentrates on the phage. The ATPase domain of the T4 terminase large subunit Gp17 is composed of two subdomains: large subdomain I and small subdomain II, which form a groove that binds to ATP (Figure 4D,E). The subdomain I structure contains a typical Rossmann fold, which contains six parallel β-folded chains surrounded by α-helices—a classical structural motif in nucleotide-binding proteins [65,66,67].

### 4.2. ATPase Functions of the Terminase Large Subunit

The amino acid sequence of HSV-1 pUL15 shares homology with the large subunit of the terminase complex of the family *Herpesviridae* and bacteriophage, particularly with respect to the two nucleotide-binding motifs in the ATP-binding domain known as Walker A and Walker B. The Walker motifs of pUL15 and its homolog are very similar in spatial structure, position in the amino acid sequence, and distance between the two motifs (Figure 3) [48,68]. The classic Walker A and Walker B sequences are G/A-4X-G-K-T/S and G-3X-L-4Z-D-E, respectively. “X” can be any amino acid, and “Z” represents a hydrophobic amino acid [69]. The Walker A can bind to ATP to cause a change in the conformation of the terminase subunit, resulting in tighter binding between DNA and ATP. These two motifs are studied more thoroughly in the phage. Take the Walker motif study of the phage as an example. In Walker A, the Gly residue is a key site for binding to ATP that also has the function of stabilizing Mg-ADP, and its inactivating mutation will lead to the reduction or even loss of enzyme activity. The Glu residue in the Walker B motif is the catalytic site of the ATPase, and its mutation will result in a complete loss of DNA packaging activity [53,54,70].

pUL15 and its homolog also have a C motif that is one of the typical features of ATPase. The C motif is an ATPase-coupled motif consisting of three amino acid residues, and the third amino acid is the most conserved and is usually a Thr or Ser residue [70] (Figure 3). The C motif mutant of T4 Gp17 is characterized by a loss of nuclease and ATPase activity and resistance to DNA translocation in vitro [70].

The amino acid sequence analysis reveals that herpesvirus terminase large subunit is a candidate for coupling the energy from ATP hydrolysis to DNA translocation, as demonstrated by the function of the large subunit of the phage T4 Gp17 [55,70,71]. The difference between the two homologs is that T4 Gp17 has weaker ATPase activity, and this activity can be activated by more than 50-fold in the presence of the terminase small subunit Gp16 [71,72]. The ATPase domain of Gp17 also displays DNA binding functions, which may be related to its involvement in the packaging process of DNA [73]. However, in HCMV, although the pUL89 has the Walker A and Walker B motifs, it does not exhibit any ATPase activity; interestingly, the ATPase activity of pUL56 has been reported [74,75]. In addition, pUL89 can enhance the ATPase activity of pUL56 [75,76].

### 4.3. Nuclease Functions of the Terminase Large Subunit

**Figure 5:**
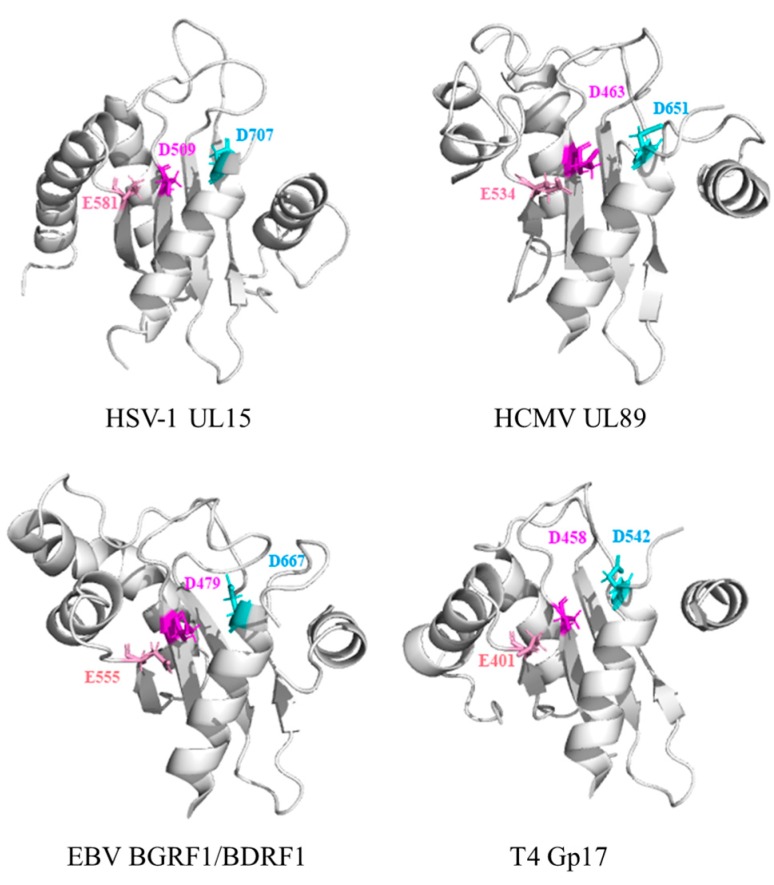
Comparison of the terminase large subunit active site region with those of HSV-1 pUL15, HCMV pUL89C, EBV BGRF1/BDRF1, and T4 Gp17, showing conserved features and structural variations. The conservative residues of the active site were marked. The protein structure models were produced using the I-TASSER server.

Biochemical data and structure analysis of a C-terminal domain of the HCMV terminase large subunit pUL89 revealed that pUL89 carries an RNase H-like nuclease activity that may be important for the cleavage of viral concatemeric DNA into monosomic genomes [57]. The nuclease activity of the protein is activated when the DNA packaging process begins and ends, and then pUL89 binds and cleaves the long concatemeric DNA into unit-length genomes for encapsidation [57]. This activity is enhanced when pUL56 is present [75]. In HSV-1, the viral DNA cannot be normally cleaved and packaged when the conserved amino acids are mutated at positions Asp509, Glu581, and Asp707 in the pUL15-C nuclease active site, corresponding to residues Asp463, Glu534, and Asp651 in HCMV pUL89C; Asp479, Glu555, and Asp667 in EBV BGRF1/BDRF1; and Asp401, Glu458, and Asp542 in T4 Gp17, respectively (Figure 4 and Figure 5) [23,57]. These three sites constitute a metal binding cluster in the nuclease domain, which is necessary for large subunit nuclease activities [56]. Metal ions can maintain the structure of the active center of the enzyme molecule and the correct structure of the complex formed by the active center and the substrate. Different viruses have different preferences for divalent cations. For example, HCMV UL89 requires Mn 2+ [57,61], HSV-1 UL15 requires Mg2+ [50], and T4 Gp17 nuclease also requires Mg2+ as a prosthetic group [66,77]. Interestingly, in vitro experiments revealed that pUL15 and its homologous proteins showed nonsequence-specific nuclease activity [50].

It is worth mentioning that in HSV-1 mutants, which lack the 400–420 amino acids between the ATPase and nuclease domains in pUL15, the results showed that the DNA cleavage is slightly defective but the DNA packaging efficiency is greatly reduced [22]. In the T4 phage, the ATPase domain and the nuclease domain are connected by a "hinge". A study has shown that ATPase and nuclease activity can proceed normally when the two domains are expressed alone; however, DNA transfer activity is completely lost [77]. These results indicate that the ATPase and nuclease domains of pUL15 are in different regions of the protein, respectively, but the region between the two domains also plays an important role in the packaging of viral DNA.

## 5. Three Models of the Terminase Complex Entering the Nucleus

### 5.1. Nuclear Import of Viral Protein

Studies have shown that the entry of the majority of viral proteins into the cell nucleus requires the help of a cellular nuclear transport mechanism [78,79,80,81]. The nuclear localization signal (NLS) of the cargo protein interacts with the importins in the cytoplasm to form a cargo–importin complex that eventually enters the nucleus through the nuclear pore complex (NPC) on the nuclear membrane [82,83]. Cargo proteins can interact directly with importin α or be mediated by transporter β and snurportin (SNP) [84,85,86,87].

The classic NLSs are generally divided into two types: monopartite and bipartite NLSs. Monopartite NLS is a short peptide, composed of four to eight amino acids rich in basic amino acids, and generally contains four or more Arg and/or Lys. Both ends of monopartite NLS are usually acidic amino acids or Pro or Gly, and the core sequence is expressed as (K/R) X (K/R). The bipartite NLSs are generally composed of two clusters of basic amino acid residues, which are separated by 10–12 nonconservative amino acid residues. The core sequence is represented as R/K(X) 10–12 RRKK [88,89].

### 5.2. NLS of the Terminase Large Subunit Mediates HSV-1 Terminase Complex Entry into the Nucleus

In HSV-1-infected cells, pUL15, pUL28, and pUL33 initially form a complex in the cytoplasm and enter the nucleus at the late stage of infection [20,34,35]. In transiently transfected cells, HSV-1 pUL15 displays nuclear localization when it is expressed alone, while pUL28 and pUL33 remain in the cytoplasm. However, pUL28 and pUL33 can enter the nucleus when coexpressed with pUL15 [90], suggesting an interaction among the three proteins and pUL15 plays an important role in the localization of pUL28 and pUL33 in the nucleus. Further analysis indicated that HSV-1 pUL15 contained a classic NLS at amino acids 183–189 (PKKRAKV). Mutants lacking a pUL15 NLS region will localize exclusively in the cytoplasm. The pUL15/pUL28/pUL33 complex also exclusively localizes to the cytoplasm of cells infected with a mutant virus lacking the pUL15 NLS [35]. These data indicate that pUL15 NLS is necessary for the nuclear localization of pUL15 and the terminase complex. In eukaryotic cells, NLS can bind to nuclear transport receptors, allowing NLS-containing proteins or their complexes to enter the nucleus through NPCs [91,92].

### 5.3. NLS of the Terminase Small Subunit Mediate Terminase Complex Entry into the Nucleus

**Figure 6:**
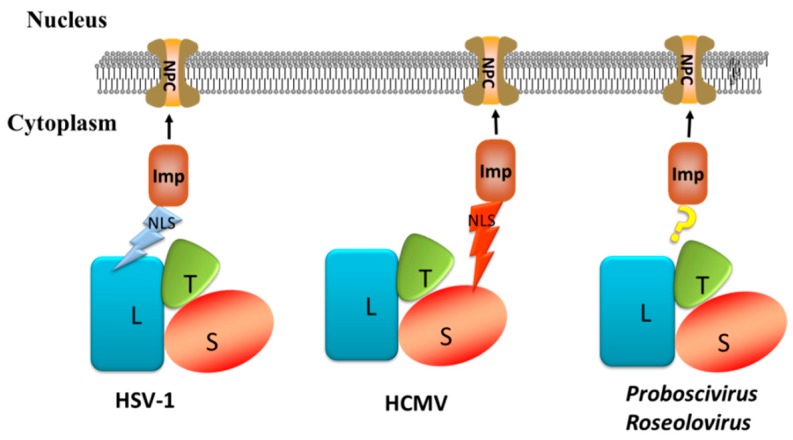
Schematic diagram of terminase nuclear transport modes for different types of viruses. L represents terminase large subunit, S represents terminase small subunit, and T represents terminase third subunit. Terminase binds to the importins and enters the nucleus through the nuclear pore complex (NPC).

Biological analysis of the HSV-1 pUL15 NLS showed that the strong NLS in pUL15 gives pUL15 the ability to enter the cell nucleus independently. However, this presentation is only in the α-herpesvirus and not in the β- and γ-herpesviruses (Figure 6) [88,89].

In some viruses, strong NLSs are detected in other terminase subunits instead of the NLS in the pUL15 homolog proteins [93]. As with the β-subfamily HCMV, pUL89 was exclusively cytoplasmic when expressed alone. This finding is consistent with the results of a bioinformatics analysis showing that there is no strong NLS in the pUL89 amino acid sequence. However, the percentage of cells with pUL89 nuclear staining increased to 26% in the presence of terminase small subunit pUL56, which contains NLSs at amino acids 816–827 (RRVRATRKRPRR), and to 80% when all three subunits (pUL89, pUL56, and pUL51) are present [93,94]. Studies have shown that pUL56 is able to enter the nucleus independently, and its nuclear transfer activity is mediated by the NLS–importin pathway [93].

### 5.4. Holoenzyme Mediates Terminase Complex Entry into the Nucleus

The terminase subunits of other viruses, such as *Proboscivirus* and *Roseolovirus*, do not possess strong NLSs that allow each protein to enter the nucleus independently, but they can be transported into the nucleus in the presence of the tripartite terminase complex [91]. Some researchers hypothesize that there may be a part of the NLS in each subunit of the terminase complex in the γ-herpesviruses. When all subunits form a complex, the complete NLS is activated [91].

## 6. Interaction between the Terminase Large Subunit and Other Viral Proteins

In herpesviruses, three subunits of terminase form a complex through their interactions which is significant for their stability in cells. In HCMV, after transfection of cells with HCMV bacterial artificial chromosome (BAC) lacking UL51, UL56, or UL89 open reading frames (ORFs) in the cells, respectively, the expression levels of the remaining terminase subunits will be decreased [95]. In addition, pUL51 stabilizes pUL56 and pUL89 expression levels and enhances pUL56–pUL89 interaction [96]. This property is not limited to HCMV pUL51 but also applies to other herpesviruses. According to reports, the same phenomenon is also present in HSV-1 pUL15, pUL28, and pUL33, and the terminase subunits are also stabilized with each other [97,98]. In HSV-1, pUL15 interacts mainly with the C-terminus of the pUL28, and the interaction between pUL33 and pUL15 is mediated by pUL28. The interaction between pUL33 and pUL28 enhances the interaction between pUL28–pUL33 and pUL15 [98,99].

The portal protein is embedded in the surface of the capsid and forms a channel structure that mediates DNA entry into the shell. The portal interacts with the terminase large subunit and is a docking site for the terminase complex on the capsid [100]. A recent study showed that there is a novel fivefold symmetrical assembly between the portal protein and the portal-vertex-associated tegument (PVAT) in HSV-1, and each subunit of the pentamer has a well-resolved two-helix coiled coil extends vector [101]. Since HSV-1 pUL33 is predicted to be largely α-helical in structure [21], there is the possibility that this pentamer is the third subunit of the terminase, pUL33. This assumes that the terminase interaction at the fivefold symmetrical portal vertex is achieved by the third subunit pUL33.

## 7. Antiviral Inhibitors Targeting the Terminase Large Subunit

### 7.1. Antiviral Drugs Targeting Herpesviruses

Herpesvirus is highly transmitted in nature and has been gradually recognized by humans as a virus related to human diseases [102]. The World Health Organization (WHO) reports that approximately 90% of the world’s population is infected with the herpesvirus, and at least nine herpesviruses can infect humans [102,103]. Many drugs that inhibit the replication of herpesvirus target viral DNA polymerase [104,105,106,107,108]. Since these antiviral drugs share a common goal, the incidence of cross-resistance should also be considered [109]. Long-term use of these drugs may lead to the emergence of drug-resistant strains [110,111]. In addition, due to the presence of blood toxicity and nephrotoxicity in some drugs, they may not be a good choice for pregnant women [112,113]. Considering these issues, it is necessary to explore new antiviral drugs and constantly search for new and highly effective antiviral targets. The DNA packaging process of herpesvirus does not have a counterpart in eukaryocyte cells. The development of drugs targeting the DNA packaging process of herpesvirus is safer and more reliable, and the terminase is a good target. So far, many drugs that target herpesvirus terminase are against HCMV.

### 7.2. Small Molecular Inhibitors Targeting the HCMV Terminase Large Subunit

Currently, the three main drugs used for the treatment of HCMV (ganciclovir, cidofovir, and foscarnet) target the DNA polymerase pUL54 [106,107]. With the emergence of cross-resistance of antiviral drugs, there is a need to continuously develop new antiviral drugs. The benzimidazole D-ribonucleoside BDCRB is a potent inhibitor of HCMV replication [114,115]. BDCRB is a nucleoside analog that can inhibit the ATPase activity of the terminase small subunit pUL56, while a high concentration of BDCRB can partially inhibit the nuclease activity of UL89 [76,116]. Two resistant HCMV strains were analyzed and found to have resistance mutations in pUL89 (specifically, D344E and A355T) and pUL56 (specifically, Q204R) [117]. The antiviral effect of BDCRB may be achieved by interfering with the binding of the terminase to DNA. This drug may also inhibit the formation of the terminase structure (i.e., the drug will inhibit the interaction between pUL89 and pUL56). BAY 38-4766 is a nonnucleoside inhibitor for the cleavage and packaging of the HCMV genome. Sequence analyses of HCMV strains that are resistant to BAY 38-4766 indicate that pUL89 and pUL104 are likely targets [118]. Wang et al. found that hydroxypyridonecarboxylic acids (HPCAs) feature a chelating motif that interacts with pUL89 in an Mn2+-dependent manner at the active site, prevents pUL89-C endonuclease activity, and inhibits HCMV replication and genome cleavage [119]. Another compound, GW275175X, is a derivative of D-ribopyranose of BDCRB. It was found that the corresponding mutation, pUL89 D344E amino acid substitution, also appeared in the strain resistant to this drug [120]. It is speculated that its antiviral mechanism is similar to that of BDCRB.

There are also some drugs that target the terminase small subunit. Recently, the terminase inhibitor letermovir has been approved for the prevention of HCMV infection caused by hematopoietic stem cell metastasis. A recent Phase III trial showed that the use of letermovir can significantly reduce the risk of HCMV infection [121]. Letermovir inhibits the packaging of viral DNA into the capsid [122]. Analysis of HCMV strains with a high level of resistance to letermovir revealed that several mutations occurred in the terminase subunit pUL56 (V236M, L241P, C325Y, R369M/G/S) [123]. In addition, there is no bone marrow toxicity or nephrotoxicity in the use of letermovir [121].

## 8. Conclusions

The replication process of herpesviruses includes adsorption, penetration, shelling, biosynthesis, assembly, maturation, and release. The assembly is a virus-specific process that can be used as an antiviral drug target. Studies have shown that the packaging process of dsDNA viruses is mainly accomplished by pUL6, terminase complex, pUL17, pUL25, and pUL32 or their homologs [16,18,124,125].

The terminase complex generally consists of a large subunit, a small subunit, and a third subunit. The large subunits possess two conserved nucleotide-binding motifs—Walker A and Walker B motifs. These two motifs have ATPase activity and allow the terminase large subunit to hydrolyze ATP, providing energy for genome translocation. Studies have shown that the conserved Walker motifs of the terminase large subunit have an important influence on DNA transfer and virus replication in HSV-1and lambda phage [53,126]. In addition, the terminase large subunit possesses nuclease activity, ensuring that tandem genomes are cleaved and a complete unit genome is inserted into each viral procapsid. The ATPase and nuclease activities of the terminase large subunit are essential functional enzymatic activities for the virus genome packaging process [48,65,127].

The nucleocapsid assembly process of the herpesvirus occurs mainly in the nucleus, so the process of transporting terminase complex into the nucleus is necessary. The terminase is a protein complex molecule larger than 200 kDa, and its nuclear entry depends on the active transport of the system [23,128]. The protein that enters the nucleus by active transport must have a special signal in its sequence, known as NLS. The way that entry into the nucleus occurs is between different herpesvirus terminases. The terminase large subunit pUL15 of HSV-1 and the terminase small subunit pUL56 of HCMV all contain NLS, but the presence of NLS is not detected in the terminase of some viruses, such as γ-herpesvirus and some β-herpesviruses [91]. The nuclear import mechanisms of terminase should be further studied.

Notably, the viral DNA encapsidation mechanism is not present in mammalian cells, so the proteins involved in this process show promise as targets for the development of antiviral drugs, which are relatively safe and reliable. Studies have reported that some DNA packaging inhibitors of the herpesvirus specifically target HCMV pUL89 and pUL56 [119,123,129,130]. These findings will be helpful for the prevention and treatment of herpesvirus.

## Figures and Tables

**Table 1:** Features of herpesvirus UL15 gene and homologs.

Subfamily	Virus Name	Abbreviation	GenBank Accession Number	Coding Region	Exon/Intron	Number of Amino Acids
*Aphaherpesvirus*	Herpes simplex virus 1	HSV-1	AER38023.1	UL15	2/2	735
	Varicella-zoster virus	VZV	NP_040165.1	ORF42	2/2	747
	Macacine alphaherpesvirus 1	MaHV1	ARS02909.1	UL15	2/2	738
	Cercopithecine alphaherpesvirus 2	CeHV-2	YP_164457.1	UL15	2/2	735
	Papiine alphaherpesvirus 2	HVP-2	AHM96136.1	UL15	2/2	735
	Felid alphaherpesvirus 1	FHV-1	YP_003331564.1	UL15	2/2	734
	Suid alphaherpesvirus 1	SuHV-1	YP_068358.1	UL15	2/2	735
	Bovine alphaherpesvirus 5	BoHV-5	YP_003662508.1	UL15	2/2	737
	Bovine alphaherpesvirus 1	BoHV-1	APW77369.1	UL15	2/2	735
	Equid alphaherpesvirus 4	EHV-4	NP_045262.1	ORF44	2/2	734
	Equid alphaherpesvirus 1	EHV-1	BAM75895.1	ORF44	2/2	734
	Meleagrid alphaherpesvirus 1	MeHV-1	NP_073308.1	HVT022	2/2	738
	Duck enteritis virus	DEV	YP_003084405.1	UL15	2/2	739
*Betaherpesvirinae*	Human cytomegalovirus	HCMV	YP_081537.1	UL89	2/4	674
	Murid betaherpesvirus 1	MuHV-1	CCE56594.1	M89	2/5	678
	Human betaherpesvirus 6A	HHV-6A	APO38446.1	U60	2/4	666
	Macacine betaherpesvirus 3	MaHV-3	AAT07420.1	grh124	2/5	671
*Gammaherpesvirinae*	Epstein–Barr virus	EBV	YP_401690.1	BGRF1/BDRF1	2/4	690
	Bovine gammaherpesvirus 4	BoHV-4	AEL29773.1	ORF29	2/4	682
	Murid gammaherpesvirus 4	MuHV-4	AAF19294.1	29	2/4	679
	Equid gammaherpesvirus 2	EhV-2	NP_042630.2	ORF29	2/4	686

**Table 2:** Catalytic site of nuclease activity in pUL15 and homologs.

Active Sites of Catalytic Domain	HSV-1 pUL15	HCMV pUL89	EBV BGRF1/BDRF1	T4 Gp17
Asp	509	463	479	401
Glu	581	534	555	458
Asp	707	651	667	542
